# Consumers’ acceptance toward whole and processed mealworms: A cross-country study in Belgium, China, Italy, Mexico, and the US

**DOI:** 10.1371/journal.pone.0279530

**Published:** 2023-01-11

**Authors:** Daylan Amelia Tzompa-Sosa, Roberta Moruzzo, Simone Mancini, Joachim Jietse Schouteten, Aijun Liu, Jie Li, Giovanni Sogari

**Affiliations:** 1 Department of Food Technology, Safety and Health, Food structure and Function Research Group, Faculty of Bioscience Engineering, Ghent University, Gent, Belgium; 2 Department of Veterinary Sciences, University of Pisa, Pisa, Italy; 3 Interdepartmental Research Center Nutrafood “Nutraceuticals and Food for Health”, University of Pisa, Pisa, Italy; 4 Department of Agricultural Economics, Ghent University, Gent, Belgium; 5 China Center for Food Security Studies, Nanjing Agricultural University, Nanjing, China; 6 Charles H. Dyson School of Applied Economics and Management, Cornell University, Ithaca, New York, United States of America; 7 Department of Food and Drug, University of Parma, Parma, Italy; Wroclaw University of Environmental and Life Sciences: Uniwersytet Przyrodniczy we Wroclawiu, POLAND

## Abstract

The interest in edible insects as food is growing, both in traditional and non-traditional insect-eating countries given their advantages in terms of sustainability and nutritional content. However, only a few studies have conducted cross-country investigations on the acceptance of including processed or whole insects in the diet. Thus, this study aimed to examine to which extent consumers were accepting (i) whole and visible mealworms, (ii) processed mealworms in their diet and (iii) to explore the factors affecting the acceptance level of consuming mealworms in countries with and without entomophagy tradition. An online survey was applied to collect responses (3,006) from five countries–i.e., Belgium, China, Italy, Mexico, and the US–using a quota sampling method. Moreover, an information treatment was included with about half of the participants receiving information about the advantages of edible insects as food (ingredient) and the presence of food safety regulations. Across countries, gender was the main factor affecting acceptance level as men accepted mealworms more than women. Entomophagy tradition mainly explained the differences among countries. Countries with entomophagy traditions (Mexico and China) showed higher acceptance of including whole or processed mealworms in the diet compared to countries with no entomophagy traditions (i.e., Belgium, Italy, and the US). While information and age did affect differently the acceptance of including processed mealworms in countries with entomophagy traditions showing that consumer acceptance was affected by information in Mexico and by age in China. Whereas it was found that younger people (below 42 years old) in countries without entomophagy tradition were more open to accepting processed mealworms in their diet. Moreover, across countries, the acceptance of including processed mealworms was higher compared to whole mealworms. These findings provide insights into which consumer segments to target and the potential impact of information when introducing new insect-based foods in countries with and without entomophagy traditions.

## Introduction

The growing world population puts additional pressure on food security and the environment. This situation has urged the search for novel protein sources that can be produced under sustainable food production systems. As such, there is a growing interest in the use of edible insects, either as whole insects or as food ingredients, as they are considered more sustainable alternatives to traditional animal-based food sources [1, 2]. Although insect production has been depicted as sustainable, there are differences among insect species in terms of feed conversion rates and the energy expenditure greatly varies among farm locations. For instance, the feed conversion rate (FCR) of mealworms (*Tenebrio molitor*, FCR of 3.8–5.8) is higher than for black soldier fly larvae (*Hermetia illucens*, FCR of 1.4–2.6) and can be similar to that of pigs (FCR of 3.1); hence, mealworms are not as efficient in converting feed into body weight compared to black soldier fly larvae. In addition, the energy expenditure for mealworm production could be higher than that of milk or chicken if produced in cold regions [2–4]. However, when a full life cycle assessment analysis is performed, mealworm production is a more sustainable alternative protein source than milk, chicken, pork, and beef since it uses less land and produces fewer greenhouse gases [3]. Moreover, mealworms can be fed using food by-products supporting the idea that edible insects could close the agri-food cycles from a circular economy point of view [5]. Also, from a nutritional standpoint, mealworms contain high-quality components, e.g., around 17% of proteins and n-3 fatty acids [2, 6, 7]. Several micronutrients in mealworms, such as iron, vitamin D, vitamin A, vitamin E, and zinc, make the inclusion of insects in the human diet a strategy to tackle common micronutrient deficiencies [2–4, 8–10].

Mealworms, like other edible insects, are not part of the traditional diet in many countries, especially in the Global North [11]. Meanwhile, entomophagy (the practice of eating insects) is widespread in several countries around the globe [12]. In Mexico, Ramos-Elorduy and Viejo Montesinos [13] reported the commercial production of mealworms as food. However, in 2020, there was only one legally registered farm in Mexico producing mealworm larvae as food [14]. Moreover, the consumption of mealworms is not generalized across Mexico. In this country, there is no legislation regulating insect production, processing, or trade [14], and edible insects are sold without control in urban markets or via small sales points [15]. In China, mealworms were initially introduced as bird feed. Nowadays, they are farmed in many regions and they are processed into food products like snacks [16]. In this country, there are no enacted national laws or standards related to edible insects; however, some local and provincial standards for edible insects exist, namely ‘T/CSCC 001–2021 Insect tea (Substitute tea)’, a tea product fermented by insects, and ‘DBS45/ 030–2016 Food safety local standard, for edible frozen fresh silkworm pupae’ [17, 18]. At a global level, there has been a growing interest in mealworm commercialization due to the aforementioned reasons related to sustainability and food security. A recent study showed that 30 products have been launched between 1996–2021, especially using mealworm powder for producing meat snacks and energy bars [19]. This interest in European countries can be also explained by the two positive scientific opinions issued by the European Food Safety Authority (EFSA) in November 2020 [20] and July 2021 [21] about the use of mealworms as novel food. This insect species has been approved to be used in different food applications, namely as a whole, as a dried insect in the form of snacks, and as a food ingredient [22]. In the US, there is no specific set of standards by the Food and Drug Administration (FDA), which has no regulatory framework but opined that edible insects are considered as food if they are specifically raised to be used for food or as components in food [23]. Interestingly, we are experiencing a decline in insect consumption in traditional Eastern countries (e.g., China) due to primary changes in lifestyle (globalization) and increased food varieties and diversified diets adopted from Western countries [24]. Therefore, reasons to accept or reject the introduction of food like edible insects in the diet could be significantly different based on the country of investigation. Nevertheless, consumer acceptance has been recognized as one of the most important to entomophagy [25]. It is important to highlight that in general accepting a new food is not simply “try” something new but rather to integrate it into their eating habits [26]. However, no common definition exists on consumer acceptance and several authors measured this variable as the willingness to pay, intention to try or purchase a product [27, 28]. Regardless this different perspective, the concept of food acceptance is influenced by different factors such as familiarity with the product, risk and benefit perceptions, sensory attributes, the information provided, as well as the influence of individual characteristics and social context [26, 27, 29, 30]. Moreover, there are universal traits recognized to be important factors to accept a novel food. One of these is referred to as neophobia which is the individual tendency to avoid a new food generally due to feelings of fear and refusal. In fact, consumers’ acceptance studies have been mostly conducted on novel foods and technologies such as gene technology (e.g. Genetically modified food), and more recently cultured meat, algae, and edible insects [27, 31].Currently, only a few studies have conducted cross-country investigations on consumer acceptance and attitudes towards edible insects. Hartmann *et al*. [32] showed that Chinese consumers have more favorable attitudes towards insects than Germans. Moreover, the German population was more willing to eat products with processed insects (i.e., used as ingredients reducing the visibility of the insects) than whole insects. Using a focus group approach Tan *et al*. [33] found that cultural exposure determines which items are recognized as food by Dutch and Thai consumers. In contrast with Hartmann *et al*. [32] and Tan *et al*. [33] found that reducing the visibility of insects did not necessarily improve the liking of a product. Other studies used pictures of several edible insect food products as a stimulus to investigate consumers’ acceptance. The main results showed that acceptance towards food containing insects increases when the visibility of insects decreases and when the products are appealing to consumers [34, 35].

Processing insects to make them invisible in food is seen as the most important single strategy to promote positive appearance evaluations [1, 25], thus, more research is needed to examine to which extent consumers are willing to accept either whole or processed insects. Also, there are significant differences within the European Member States. For instance, Menozzi *et al*. [36], found that Dutch consumers have a higher intention to eat products containing processed insects than Italians. These contrasts probably originate from the different food cultures, e.g., a strong gastronomic tradition in Mediterranean countries vs cosmopolitan and international cuisine in northern European countries. Furthermore, it is unclear the extent to which socio-demographic differences, including gender, age, and entomophagy tradition, would affect the acceptance of this novel food [37]. Finally, as recommended by Ruby and Rozin [38], there is a lack of research on representing the largest countries (e.g., China) and countries where insect consumption is normalized (e.g., Mexico and China) [15].

Therefore, this study aimed to examine to which extent consumers are willing to accept mealworms as food, either whole and visible or processed. The study included five countries (Belgium, China, Italy, Mexico, and the US) with high and low entomophagy traditions. The acceptance to include whole and processed mealworms in the diet was evaluated based on their country of origin, their gender (male vs female), their age (18–41 years old vs 42 years old and older), and the macro-region within a country (e.g., North vs South area). We also measured whether providing information about (i) the advantages in terms of sustainability and nutrition of using edible insects as food and (ii) presence of food safety regulations would have an impact on their acceptance. The results of this study could be of interest for scholars exploring consumer acceptance and attitudes towards insects as food as well as for practitioners in the food industry to develop appropriate marketing campaigns.

## Materials and methods

### Schematic overview of survey program

**Fig 1:**
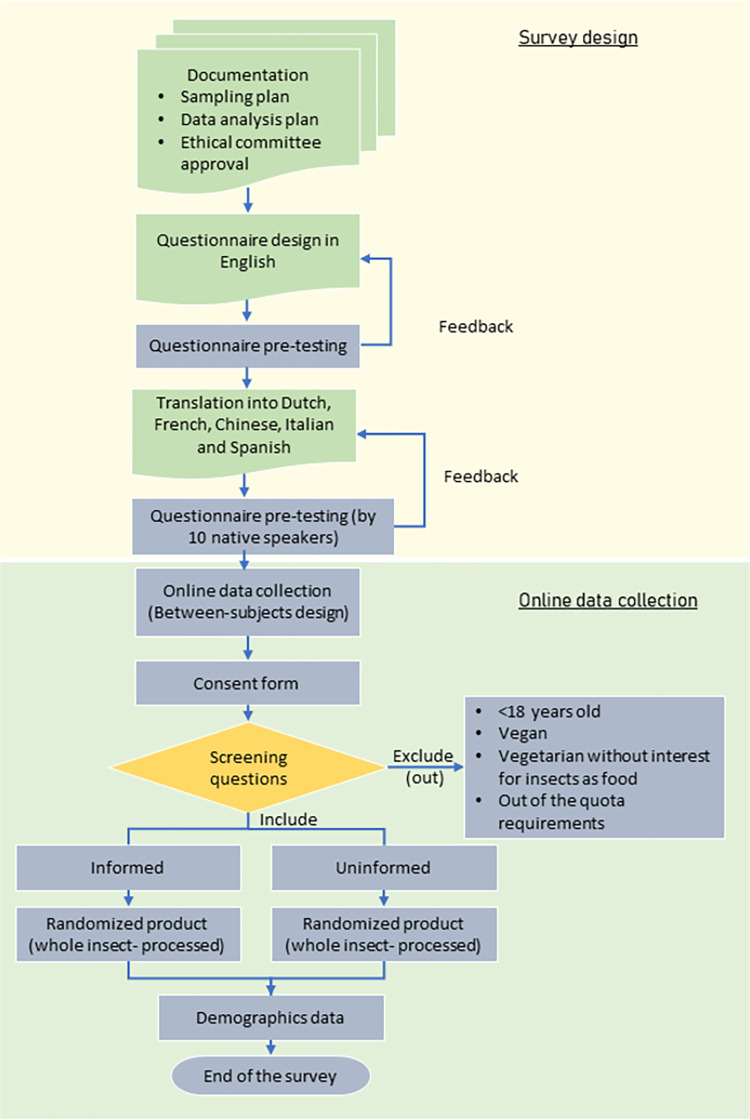
Schematic overview of the survey design and data collection of this study.

Fig 1 shows the schematic overview of the survey design and data collection. Initially, the questionnaire was designed in English and was pre-tested by experts in consumer science. After approval, the questionnaire was translated into Dutch, French, Chinese, Italian and Spanish. The translated surveys were pre-tested by a minimum of 10 subjects with residence in the respective countries under study which were unrelated to the project. These, pre-tests gave feedback to the researchers and the questionnaire was adjusted accordingly. Data collection was performed online. The various steps were guided by standard well-thought through procedures, which are reproducible and validated. Details on the survey design, questionnaire and sampling procedure can be found in the coming sections. For emphasis, this work aimed to examine to which extent consumers were accepting whole and visible mealworms; processed mealworms in their diet; and to explore the factors affecting the acceptance level of consuming mealworms in countries with and without entomophagy tradition.

### Study area and sampling procedure

An online survey was conducted in Belgium, China, Italy, Mexico, and the US between February and March 2022. The selection of the countries was done for several reasons. First, these countries represent the region where the authors currently work and therefore they are familiar in terms of population, culture, insect market. Second, the five countries represent a diversified target of consumers and the tradition of entomophagy: Third, countries were selected in order to include both regions with a strong entomophagy tradition (i.e., China and Mexico) and without an entomophagy tradition (i.e., Belgium, Italy, and the US). Moreover, each country has unique food habits and attitudes towards foods, including countries–like Italy–with a strong and long-standing gastronomic tradition [36] and others with a more international cuisine, like the US. The survey was designed using Qualtrics®, a software to collect and store data and often used in consumer studies. The questionnaire was administered by an online marketing agency that distributed the survey link to panelists. Several criteria were used to define our final sample: subjects were excluded when they were i) younger than 18 years old, ii) vegan, or iii) vegetarian without an interest in insects as food. Moreover, fast respondents (below 40% of the median time to complete the survey) were excluded to ensure high-quality data. Additionally, a quota within each country was set for gender, age, and macro-region (i.e. north, south, east, west), to ensure participants constituted a representative sample. A total of 3,006 responses were collected. The final sample per country is shown in Table 1, whereas the provinces within macro-regions in each country are reported in S1 Appendix.

**Table 1:** Sample characteristics per country under study.

Country	Total	Gender		Information treatment		Age (years old)		Macro-region^1^					
		Male	Female	Uninformed	Informed	≤41	≥42	1	2	3	4	5	6
**Belgium**	518	247	271	273	245	194	324	425	38	54	-	-	-
**China**	547	302	245	279	268	331	216	84	22	218	44	123	56
**Italy**	528	250	278	281	247	193	335	137	99	98	194	-	-
**Mexico**	715	328	387	353	362	423	292	194	295	213	-	-	-
**US**	698	353	345	368	330	331	367	262	158	122	140	-	-

^1^ For macro-regions within each country, please see S1 Appendix.

Respondents electronically approved an informed consent form before their participation. The study was granted ethical approval by the University of Pisa Ethics committee (Committee on Bioethics of the University of Pisa—Review No. 26/2021).

### Survey design and questionnaire

The survey consisted of several question groups, including general eating habits, and demographics. Following a random assignment, a between-subjects design was implemented. Approximately half of the participants received the information treatment before the question was displayed, whereas the rest of the sample represents the control group with no information. The information was adapted from the Report “Looking at edible insects from a food safety perspective. Challenges and opportunities for the sector” published by the FAO in 2021. The information provided included several elements regarding the potential benefits of using edible insects as food (sustainability and nutritional benefits), as well as the presence of food safety regulations (see S1 Appendix).

Based on the concept that consumer acceptance of insects is not only trying once the product but being willing to incorporate it into the diet, we developed two specific questions related to one of the currently authorized insect species on the market (*T*. *molitor*). Respondents were asked about their level of acceptance of including whole and processed mealworms in their diet. The two questions presented were as follows: i) “*Would you accept including whole mealworms in your diet*?”; ii) “*Would you accept including processed food (e*.*g*., *protein bar*, *pasta*, *burgers) that contains mealworm powder in your diet*?”. The two questions were divided into two separate sections of the survey to facilitate the reply.

Based on previous literature [39, 40], each closed-ended question had four possible answers: namely “*Yes*, *because…*”, “*Yes*, *but…*”, “*Maybe*, *if…*”, “*No*, *because…*” which represent four different levels of acceptance. Participants were asked to select only one option. [39, 40]. Respondents were categorized into i) millennials and generation Z (born on 1980 and after) and ii) older adults (born before 1980), thus the categorization was 18–41 years old and 42 years old or more.

### Statistical analysis

First, the effect of gender (male vs female), treatment (informed vs uninformed), age (18–41 years old vs 42 years old and more), country (Belgium, China, Italy, Mexico, and the US), product (whole vs processed mealworm), and macro-region within each country was tested using Chi^2^ test of homogeneity. When a significant difference was found (*P* < 0.05), a post hoc test (z-test) was performed to determine which of the independent variable differ in terms of the four categories of the dependent variable (“*Yes*, *because…*”; “*Yes*, *but…*”; “*Maybe*, *if…*”; “*No*, *because…*”). In the post hoc test, Bonferroni’s correction was used to correct for multiple comparisons (*P* < 0.0125) decreasing the chance of committing Type I errors. Every following comparison was performed in a similar way.

Then, the countries were divided into two groups, according to their acceptance to include mealworms in the diet, namely high (Mexico and China) vs low acceptance (Belgium, Italy, and the US). This categorization followed statistical tests between the two formed groups and within each group to check if the categorization was correct and if differences within countries in a category existed. Statistical analyses were performed using SPSS software (Version 28, IBM® SPSS® Statistics).

## Results

### Factors affecting acceptance of including mealworms within each country

Across countries, gender was the leading factor affecting the acceptance level of mealworms in the diets (Table 2). In countries with high acceptance of including mealworms as food this represented the only significant (China) or the highly significant (Mexico) factor. However, in countries with low acceptance of including mealworms in their diet (Belgium, Italy, and the US), other factors, such as information and age, did have a significant effect. In this section, we show the effects of gender (male vs female), treatment (informed vs uninformed), age (18–41 years old vs ≥ 42 years old), and macro-regions within each country on the acceptance of including mealworms in the diet of each country.

**Table 2:** Effect (*P* value) of gender, information treatment, age, and macro-region on acceptance of the inclusion of mealworms (whole or processed) in the consumers’ diet in Belgium, China, Italy, Mexico, and the US.

Country	Product	Gender	Information treatment^1^	Age^2^	Macro-region^3^
**Belgium**	Whole mealworm	0.004	0.112	0.298	0.916
	Processed mealworm	0.054	0.944	0.029	0.424
**China**	Whole mealworms	0.039	0.919	0.944	0.351
	Processed mealworm	0.851	0.986	0.085	0.919
**Italy**	Whole mealworm	0.018	0.898	0.056	0.083
	Processed mealworm	0.245	0.008	0.039	0.222
**Mexico**	Whole mealworm	< 0.001	0.364	0.184	0.040
	Processed mealworm	0.001	0.031	0.185	0.025
**US**	Whole mealworm	0.004	0.668	< 0.001	0.096
	Processed mealworm	< 0.001	0.017	< 0.001	0.072

^1^Information treatment: Uninformed vs Informed

^2^Age18-41 years old vs ≥ 42 years old

^3^See overview of macro-regions in S1 Appendix.

Significant differences assessed by Chi^2^ test of homogeneity (*P* < 0.05).

#### Gender effects within countries

**Fig 2:**
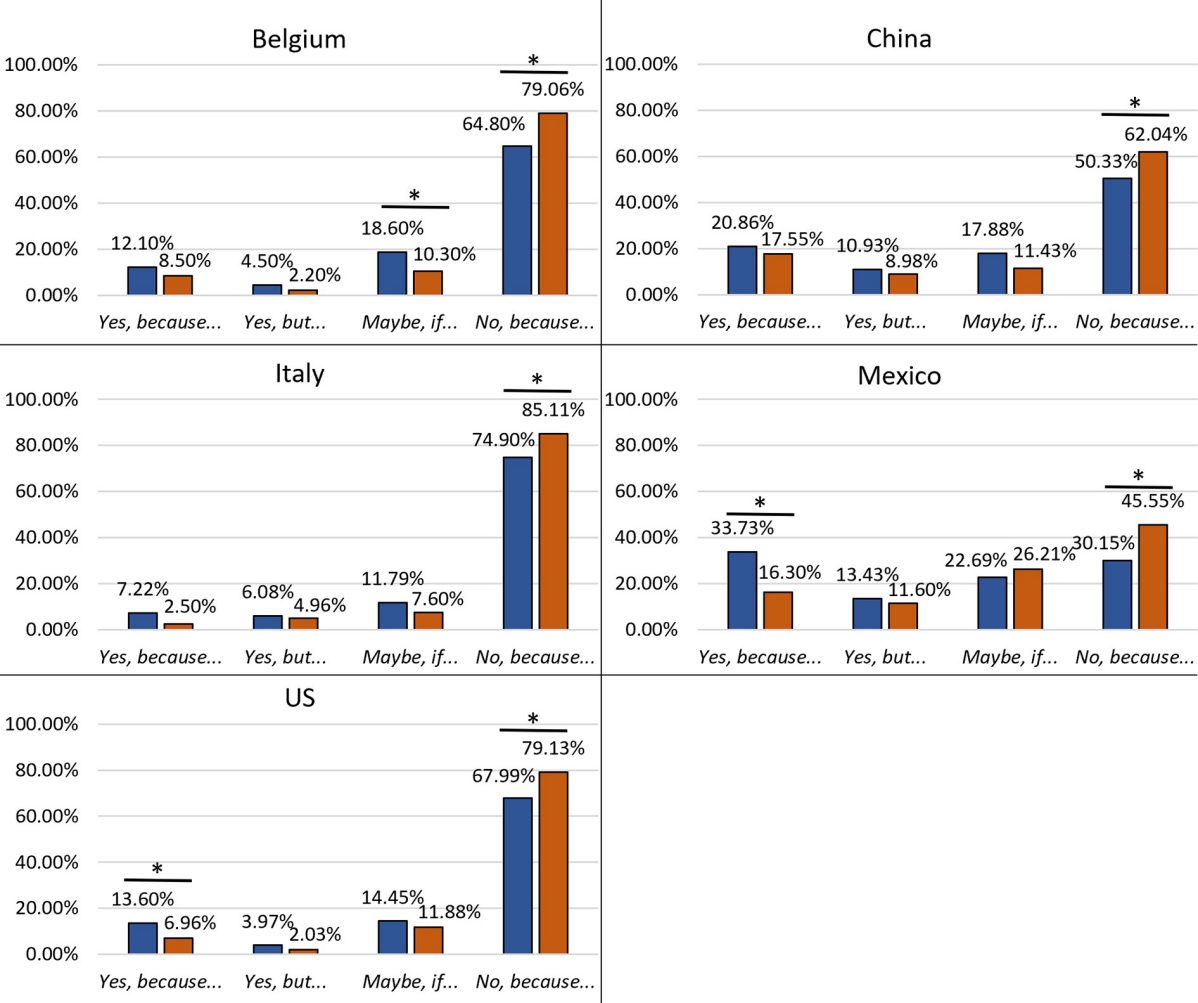
Effects of gender on the acceptance level of the inclusion of whole mealworms in the diet in different countries. Male: blue columns; Female: orange columns. *Significant difference assessed by Chi^2^ test of homogeneity with post hoc test (z-test of two proportions) with Bonferroni’s correction (*P* < 0.0125).

Males showed the lowest rejection rate (“*No*, *because…*”) towards including whole mealworms in their diet (Fig 2). The difference in the rejection rate between males and females was on average 13%, with a higher percentage in Mexico (16%, *P* < 0.001) and Belgium (15%, *P* = 0.009). Moreover, in Mexico and the US, there was a significant increase in the acceptance (“*Yes*, *because…*”; *P* < 0.001 in both countries) of whole mealworms by male participants. In Belgium, males answered “*Maybe*, *if…*” more frequently than women (+8%). Meanwhile, in Italy and China, men rejected (“*No*, *because…”*) whole mealworms less often (-10.27%, *P* = 0.004; -11.71%, *P* = 0.006, respectively) compared to women.

**Fig 3:**
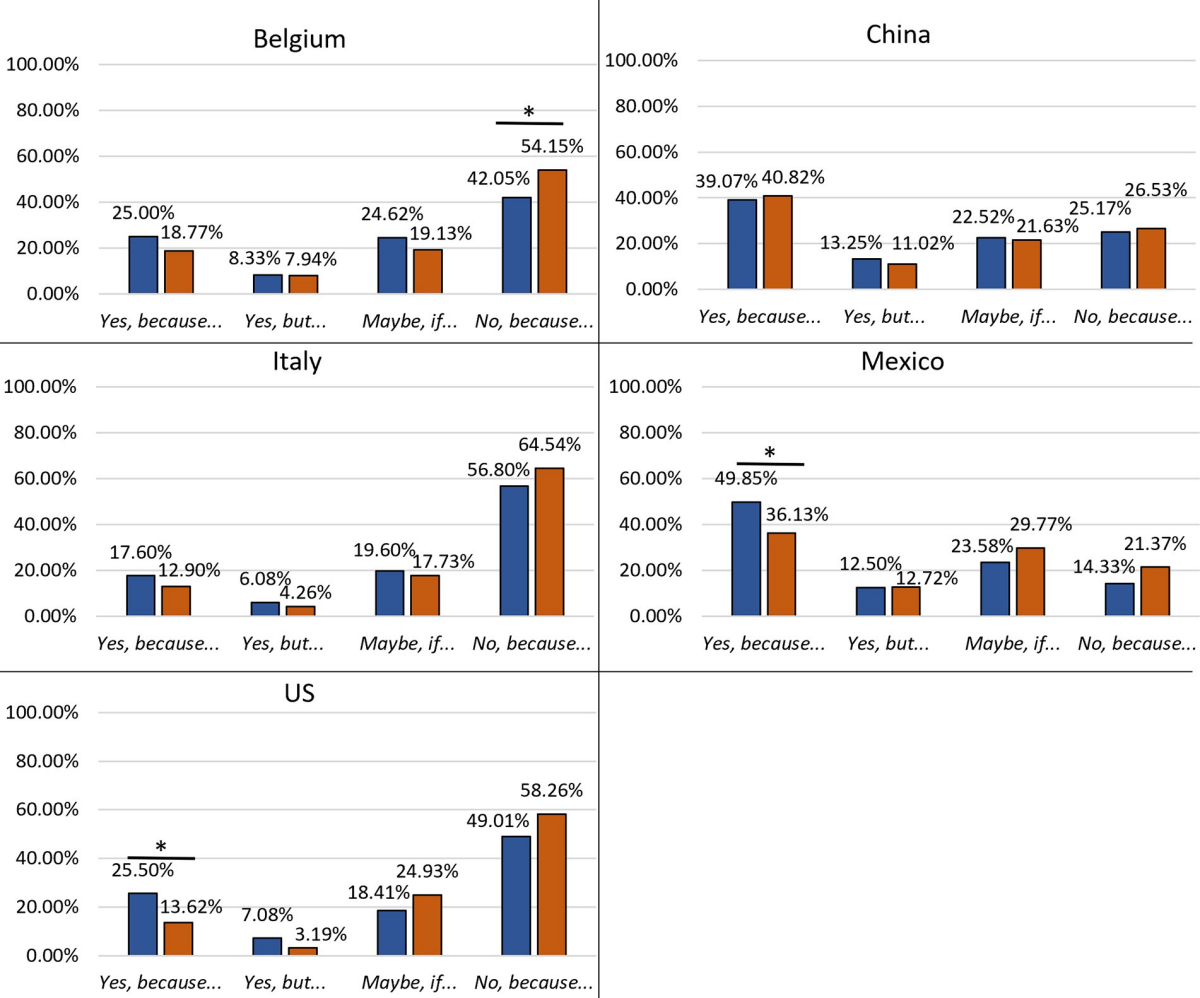
Effects of gender on the acceptance level of the inclusion of processed mealworms in the diet in different countries. Male: blue columns; Female: orange columns. *Significant difference assessed by Chi^2^ test of homogeneity with post hoc test (z-test of two proportions) with Bonferroni’s correction (*P* < 0.0125).

The effect of gender decreased when participants were asked about their acceptance of including processed mealworms in their diet. In Mexico and the US, men showed a higher acceptance level than females with an average increase of 12.5% (*P* < 0.125) (“*Yes*, *because…*”) (Fig 3). Whereas in Belgium, men rejected processed mealworms in a lower proportion compared to women.

#### Information treatment effects within countries

In the countries under study, there was no significant effect of information on the acceptance of including whole mealworms in the diet (Table 2). Regarding the effect of information on the acceptance of including processed mealworms in the diet, it affected the acceptance of participants in Mexico, Italy, and the US. However, for Mexico and the US, when the differences among acceptance levels were compared using a post hoc test and the significant difference was adjusted for multiple comparisons (Bonferroni’s correction), this significance disappeared. Only in Italy, the information given to the participants prior to the question had an effect. This effect was specific to participants doubtful about accepting processed mealworms in their diet (“*Maybe*, *if…*”). This level of acceptance increased when information about the low ecological footprint associated with insect production, their nutritional value, and information on the authorization of mealworms as food by the European Food Safety Authority (EFSA) was given to the participants.

#### Age effects within countries

**Fig 4:**
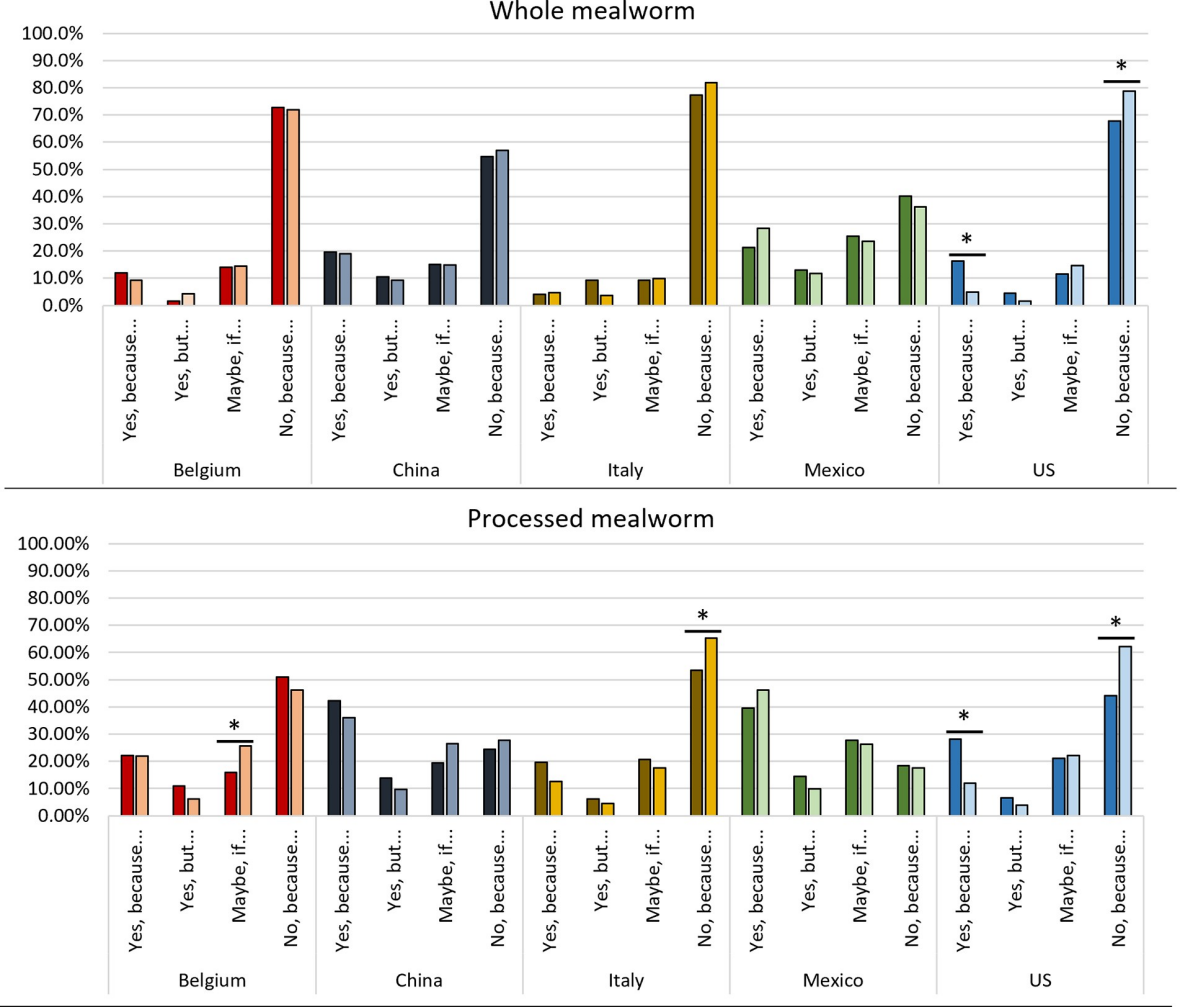
Effects of age on the acceptance level towards the inclusion of whole and processed mealworms in the diet in different countries. 41 years or younger: dark columns; 42 years or older: light columns. *Significant difference assessed by Chi^2^ test of homogeneity with post hoc test (z-test of two proportions) with Bonferroni’s correction (*P* < 0.0125).

In countries with low acceptance (Belgium, Italy, and the US), age significantly affected the acceptance of the inclusion of mealworms in the diet (Table 2). This effect was mainly seen in the acceptance of processed mealworms (Fig 4). In Italy and the US, younger participants (18–41 years old) showed a lower rejection level (“*No*, *because…*”) towards processed mealworms than older participants (≥ 42 years old) (12% and 18% less, respectively). In Belgium, older participants (≥ 42 years old) doubted more (“*Maybe*, *if…*”) about accepting processed mealworms in their diet compared to younger participants. Only in the US, age affected the acceptance to incorporate whole mealworms into their diet. In this country, younger participants (18–41 years old) showed an increase in the acceptance level of whole mealworms (+11%) and a decrease in rejection (-11%).

### Macro-region effects within countries

In the countries under study, there was no significant effect of macro-regions on the acceptance of including whole or processed mealworms in the diet, except for Mexico where a significant difference was found for both mealworm products (Table 2). However, when the differences among acceptance levels were compared using a post hoc test and the significant difference was adjusted for multiple comparisons (Bonferroni’s correction), only the significant effect for processed mealworms for *“No*, *because*…*”* remained. The region in Mexico showing the least rejection towards processed mealworms was the South/Southeast (14%) compared to the North (25%) and the Center/Center North (17%).

### Country and product effects

**Fig 5:**
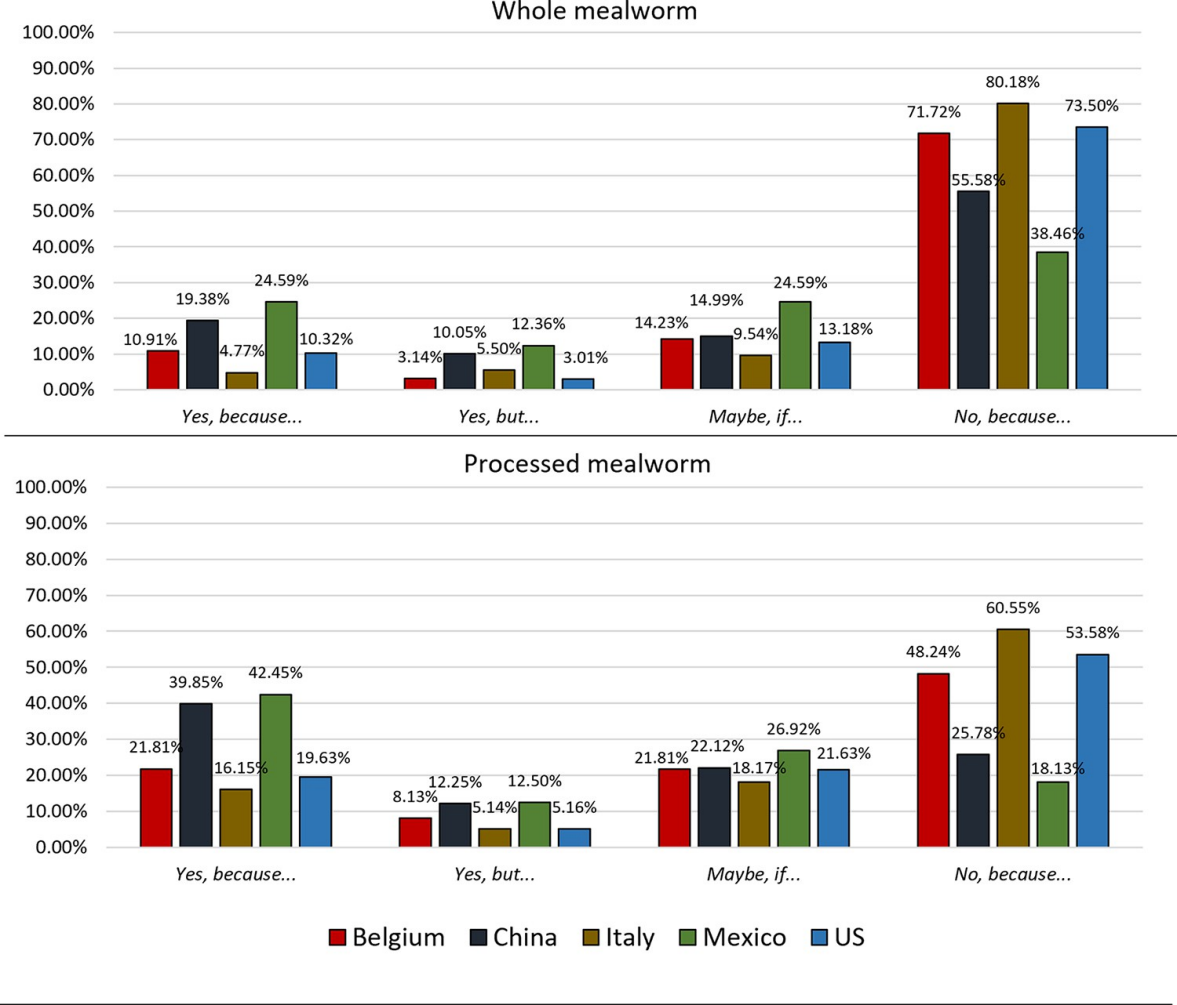
Acceptance level (%) of the introduction of mealworms, either whole or processed, into the diet of the five different countries (Belgium, China, Italy, Mexico, and the US).

The five countries involved in the study had two distinctive acceptance levels of including mealworms and processed mealworms in the diet (Fig 5), i.e., one group showed a low acceptance level and the other showed a high acceptance level.

The countries with a low acceptance level were Belgium, Italy, and the US, whereas the populations showing a high acceptance level were the Mexican and the Chinese. A strong significant difference (*P* < 0.001) between these two types of countries was found for almost every level of acceptance of including whole or processed mealworms (Table 3). The Mexicans and Chinese accepted both whole and processed mealworms at a higher level, as shown by the significantly high proportion of “*Yes*, *because…*”, “*Yes*, *but…*” and “*Maybe*, *if…*” answers compared to the other countries.

**Table 3:** Effects (*P* value) of the two mealworm products (whole or processed) on the acceptance towards inclusion in the diet.

Country	Product effect (Chi^2^ test)	Post Hoc test between whole or processed			
		*Yes*, *because*.* *.* *.	*Yes*, *but*.* *.* *.	*Maybe*, *if*.* *.* *.	*No*, *because*.* *.* *.
**Belgium**	< 0.001	< 0.001	0.001	0.001	< 0.001
**China**	< 0.001	< 0.001	0.249	0.002	< 0.001
**Italy**	< 0.001	< 0.001	0.683	< 0.001	< 0.001
**Mexico**	< 0.001	< 0.001	0.936	0.305	< 0.001
**US**	< 0.001	< 0.001	0.042	< 0.001	< 0.001

Significant differences assessed by Chi^2^ test of homogeneity followed by Post Hoc test (z-test of two proportions) with Bonferroni’s correction (*P* < 0.0125).

Comparing the countries with a high acceptance level of including mealworms in the diet, Mexican consumers showed the lowest rejection attitude of whole or processed mealworms (Table 4). Compared to the Chinese, a significantly lower proportion of Mexicans reported rejections of whole mealworms and a significantly higher proportion reported that they might (“*Maybe*, *if…*”) include whole mealworms in their diets.

**Table 4:** Effects of the country on acceptance level of the inclusion of mealworms in the diet (whole or processed).

Product	Acceptance level	Countries with a high acceptance level (China vs Mexico)	Countries with a low acceptance level (Belgium-US vs Italy)^1^	High acceptance vs low acceptance countries^2^
**Whole mealworms**	*Yes*, *because*.* *.* *.	0.041	< 0.001	< 0.001
	*Yes*, *but*.* *.* *.	0.185	0.011	< 0.001
	*Maybe*, *if*.* *.* *.	< 0.001	0.020	< 0.001
	*No*, *because*.* *.* *.	< 0.001	0.001	< 0.001
**Processed mealworms**	*Yes*, *because*.* *.* *.	0.394	0.007	< 0.001
	*Yes*, *but*.* *.* *.	0.857	0.323	< 0.001
	*Maybe*, *if*.* *.* *.	0.041	0.151	0.004
	*No*, *because*.* *.* *.	< 0.001	< 0.001	< 0.001

Significant differences assessed by Chi^2^ test of homogeneity followed by Post Hoc test (z-test) (*P* < 0.0125).

^1^Comparison between Belgium and US against Italy.

^2^China and Mexico (high acceptor countries) vs Belgium, Italy, and the US (low acceptor countries).

Regarding the countries with a lower acceptance of including mealworms, the Belgian and the American samples were similar to each other but different from the Italian one (*P* < 0.05), which showed the strongest rejection for both types of mealworm products (Fig 5). In Belgium and the US, there was a significantly higher acceptance of including mealworms in the diet (“*Yes*, *because…*”; 11%) than in Italy (5%) (Fig 5). Moreover, Italian consumers showed a significantly higher rejection of whole mealworms compared to the Belgian and American samples. The proportion of rejection in Italy was 80% of the sample, while it was between 72–73% in Belgium and the US.

Across countries, the acceptance of including processed mealworms in the diet (“*Yes*, *because…*”) increased from 6 to 17% compared to whole mealworms (Fig 5). Whereas the rejection of processed mealworms (“*No*, *because…*”) drastically decreased by an average of 20.5% compared to whole mealworms (Fig 5). Individuals indicating that they might consume processed mealworms (“*Maybe*, *if…*”) increased significantly (average +6.8%) compared to whole insects (Table 2), with a similar trend in each country under study.

## Discussion

In this study, we investigated how factors such as product, gender, information, and age, affect the acceptance of mealworms as food in several regions of the world. Some of these regions currently do not have entomophagy traditions, i.e., Belgium, Italy, and the US [11], whereas other countries in Latin America (i.e., Mexico) and Asia (i.e., China) have a long tradition of entomophagy [13–15, 28, 41, 42].

Across countries, gender was the foremost factor affecting the acceptance of including mealworms in the diet. Our results indicated that men accepted mealworms in their diet more than women (Table 2, Figs 2 and 3), which is in line with previous studies performed in several countries [25, 43–45]. However, some mixed results were reported in past studies [29]. For example, Lammers *et al*. [46] found no significant gender difference for processed products (i.e., insect burgers prepared with ground buffalo worms); whereas our results showed that men were more willing to consume whole or processed mealworms than women. Lundén *et al*. [47] indicated how the effect of gender was dependent on the insect species, with males being significantly more inclined to try ants than females, without significant gender differences for crickets. Men have a low-risk perception [37, 43], which could have driven their acceptance of whole or processed mealworms.

The results of this study showed that age affected the acceptance of mealworms as food only in countries with no entomophagy tradition (i.e., Belgium, Italy, and the US; Table 2 and Fig 4). The effect of age is not consistent among different studies [32, 45, 48]. Our results suggested that younger people (≤ 41 years old) were more open to accepting mealworms in countries without entomophagy tradition. Our findings are consistent with other studies where a high acceptance level of processed mealworms was reported in children and teenagers in European countries [48, 49]. The age effect could be explained by the curiosity of the young population toward novel food and a higher sensitivity to the topics related to food sustainability [44]. This statement is also corroborated by the results of Su *et al*. [41] who tested the Chinese consumers’ acceptance toward edible insects. In China, country where entomophagy can be traced back over 2,000 years ago, the consumption of insects was indeed positively correlated to the age (also to the educational level and the insect-related occupation), but in this case was the older responded to be more prone to eat insects. One of the strengths of the present study stands in the use of a very balanced sample in terms of age and gender based on national statistics. Therefore, our results for these two demographic variables could be generalized to the country’s population.

Overall, the results showed that informing the consumers about the benefits (nutritional and environmental) and safety of this novel food source does not change the acceptance of consuming whole or processed mealworms (Table 2), which is in line with some previous studies (e.g., Lensvelt and Steenbekkers [50]). Contrary to the findings in this study, other studies had shown that information about the nutritional and environmental benefits of insects can raise the acceptance of edible insects [29, 51, 52]. These contradicting results could be explained by the use of various formats to provide information to the participants, ranging from a full in-person seminar (e.g., Mancini *et al*. [51]) to a short text in a survey [53]. The content also differs among the previously mentioned studies (e.g., sustainable and nutritional benefits, safety, and sensory characteristics). Different approaches could lead to different information effects on the acceptance level. It is also known that consumer responsiveness to information highly depends on personal interests [29]. While safety is mentioned as an important factor to influence consumers acceptance of insects and insect-based food products [54], this study did not find any impact on safety information on the acceptance of whole and processed mealworms in the diet.

As expected, countries with entomophagy traditions (Mexico and China) showed a higher acceptance, regardless of their processing condition (Table 2 and Fig 5). In these countries, insect consumption is normalized in certain regions and is included in traditional dishes and recipes making insects a familiar food [15]. Previous literature [55] has shown that familiarity is a determinant factor promoting consumer acceptance of whole insects and insect-based food products being especially relevant for whole insects [56].

Interestingly, no difference in acceptance levels exists among macro-regions within countries. We initially hypothesized that the acceptance level would have differed within macro-regions, especially in sizable countries like China, Mexico, or the US., as differences in entomophagy tradition exist within a country. For instance, in Mexico, the South and Southeast regions are particularly rich in entomophagy traditions, followed by the central part of the country [15, 57–62]. However, this difference in entomophagy tradition did not seem to affect acceptance, except for the rejection of processed mealworms in Mexico (Table 2). In this specific case, a lower rejection rate was reported for the South, compared with the Central and Northern regions. Similar results were reported by Sato and Ishizuka [28] in two different areas of Japan, Kanto area (representative of the actual Japanese population) and Nagano Prefecture (area with a strong entomophagic culture). Although there was a higher acceptance of traditional insect meals in the locations with a strong insect culture, there was no appreciable difference in the acceptance of innovative insect foods between the two areas.

The results of the study showed there is a significant difference between Chinese and Mexican respondents, as the Mexican population had a higher acceptance of both whole and processed mealworms. These results are in accordance with a previous study where the willingness to eat an insect-based food product was tested in several countries including China and Mexico [63]. Remarkably, in the study by Castro and Chambers [63], the Mexican sample was the most willing to eat insect-based food products and was placed above other countries with entomophagy traditions, such as China or Thailand. Our results confirm that Mexican consumers have the highest acceptance levels of insect-based products among the other five countries investigated, which is understandable considering that Mexico has the largest number of edible species in the world [64]. We suggest that even though mealworms are not a common edible insect in Mexico and China, the familiarity of these consumers with edible insects [16, 59, 65] has positively influenced their acceptance level to include whole and processed mealworms in the diet. Therefore, we can assume that Mexican consumers in general have a higher acceptance level than the North American and European population (i.e., beyond the countries investigated in our study). Further studies should focus on cross-country evaluation in other Latin American and Asian countries, as well as in other world regions where edible insects are familiar, such as in the African continent. In fact, in a recent market research study, it has been reported that in the Africa continent, despite the tradition of entomophagy, no processed product with insect ingredient was launched in the last years [19]. The same study shows also the in Latin America the insect-based food industry is still absent compared to the US and Europe. This could be explained due the tradition in such countries to collect insects in the nature for domestic consumption or for being sold at local markets. Our results are starting point to support the mapping of the acceptance of edible insects as food in countries where, as of now, there is not much consumer research [15, 55].

The high acceptance level in Mexico can be explained by the fact that in this country insects are associated with a positive image since they are a delicacy sold either in local markets or served in high-end restaurants [59, 66]. In addition, insects are dishes reserved for special occasions or celebrations and are used in various preparations [15]. Furthermore, most edible insect species are highly priced [59, 66] and their cost is between 3.5 and 10 times the price of chicken or pork meat.

Our results from China are aligned with the solid entomophagy tradition of this country, which includes raising and breeding insects for human consumption, but also for medical purposes [16, 24]. Many insect species (e.g., silkworms, locusts, ants, honeybees, and their products) are commonly consumed in different regions, mostly in rural Chinese areas, and the level of acceptance is relatively high [16]. During the year, there are 20 to 30 popular species used in restaurants, including grasshoppers, silkworm pupae, wasps, bamboo insects, and stink bugs [41, 67]. However, some food safety concerns and the visibility of insects negatively impact the consumption frequency in China [54]. Therefore, even in this country, processed mealworms as an ingredient in foods could be a strategy to increase the level of acceptance. Moreover, in previous studies [65], it was shown that insect eaters have a lower disgust and higher intentions to engage in entomophagy than non-eaters.

In Belgium, the US, and Italy, the acceptance to include mealworms in the diet was significantly lower than in China and Mexico (Table 3). Our results are similar to previous cross-country studies showing that European countries or countries with no entomophagy tradition have a low degree of acceptance of insect-based food products [25, 63, 68]. In Europe and North America, food neophobia, disgust, and risk perceptions drive consumers’ rejection of accepting mealworms in their diets [11, 22, 68]. Among the countries with a low degree of acceptance, Belgians and Americans were more likely to accept mealworms in their diet compared to Italians (Table 4). One factor influencing the positive attitude in Belgium and the US could be the increased exposure to insect-based foods in the market and the media [11, 19]. In 2016, the Belgian Federal Agency for the Safety of the Food Chain (FAVV) approved 10 insect species for human consumption, which was followed by the introduction of several insect-based products in the market [45, 51]. For European countries, we also have to consider that the Novel Food regulation (Reg. (EC) 2283/2015)–which was put in force on January 1, 2018 –allows insect products only after authorization for their safety. Therefore, in the last years, Europeans have had limited exposure to insect-based foods. Furthermore, in these countries, the few insect products available are somehow expensive and not easily available [55]. As a result, it is not surprising that Italian consumers have the lowest acceptance of whole and processed mealworms, explained by a strong negative attitude towards insects as food [51].

Willingness to consume mealworms is one of the first steps toward increasing the consumption of edible insects. The first tasting should be accompanied by positive sensorial and emotional experiences, otherwise, repeated consumption will not be achieved [33, 34]. Furthermore, a positive experience tasting insect-based food triggers consumers to communicate with people who have not tasted such food. This process is called “word-of-mouth” and has been claimed as the main mechanism to increase the spread of insect-based food in countries with low acceptance levels [69]. It has also been stated that a proper food design is needed to develop attractive insect-based foods that are able to provide positive experiences for consumers [70]. Indeed, recent studies have shown that overall liking and preference for insect-based foods can be similar to or higher than regular food if insect ingredients are appropriately processed and used in a correct formulation and application [71, 72].

There are two primary limitations to our findings. First, this is a hypothetical survey and therefore we cannot ascertain whether the reported behavior would translate into a real behavior of willingness to consume whole or processed mealworms. Second, even if we used a quota sample based on national statistics, the representativeness is limited to the ones who have computer access and to the panel members of the agency, which means that they were probably individuals with higher socio-economic statuses. Future research could more carefully examine socio-demographic differences among and within countries.

## Conclusions

Interest in insects as a potential source of food has grown considerably in recent years and consumer acceptance studies towards edible insects as food is crucial to support this emerging sector. The overall aim was to investigate the main difference in the acceptance of mealworms as food across five countries, i.e., Belgium, China, Italy, Mexico, and the US considering a sample representative for gender, age, and macro-region (i.e. north, south, east, west). Significant differences related to the entomophagy culture of each country were found, i.e., consumers in China and Mexico with a long-standing entomophagy tradition reported higher acceptance of whole and processed mealworms than the other countries. Across all countries, gender affected the willingness to consume mealworms. In this regard, men tended to reject mealworms less than women, confirming previous results. Regarding age, younger consumers were more open to accept mealworms in their diet. Considering data across all countries, the acceptance of processed mealworms was higher compared to whole mealworms suggesting that the focus of the food industry should be on processed mealworms rather than whole insects.

Interestingly, from a marketing perspective, we observed how familiarity with edible insects as food in general increases acceptance of edible insect species not currently available on the market, (e.g., the mealworms in Mexico and China). In countries with no entomophagy tradition, the introduction of (food products made with) mealworm should target first young males. These findings unlock business opportunities for insect producers to introduce new insect species (whole or processed) in food products in countries with entomophagy traditions.

Further research should investigate how consumers’ acceptance changes based on edible insect species, starting from the ones recently authorized in the EU, i.e., *Locusta migratoria* (migratory locust) and *Acheta domesticus* (house cricket). Future studies should also consider how the knowledge, previous experience towards entomophagy, and the drivers in each market (i.e., traditional value, food security, sustainability, curiosity, health) could influence acceptance to consume insects.

## Supporting information

S1 Appendix Consent form, screening questions, information treatment, open questions, demographics. (DOCX)

S1 File (XLSX)
