# Supplementary material for: Consumers’ acceptance toward whole and processed mealworms: A cross-country study in Belgium, China, Italy, Mexico, and the US
Source: PLoS One. 2023 Jan 11;18(1):e0279530. doi: 10.1371/journal.pone.0279530 (PMC9833582; doi:10.1371/journal.pone.0279530)
Supplement: S1 Appendix — (DOCX) [file pone.0279530.s001.docx]

Consumers' acceptance toward whole and processed mealworms: A cross-country study in Belgium, China, Italy, Mexico, and the US

Daylan Amelia Tzompa-Sosa^1,2^, Roberta Moruzzo^2^, Simone Mancini^2,3^*, Joachim Jietse Schouteten^4^, Aijun Liu^5^, Jie Li^6^, Giovanni Sogari^7^

^1^Department of Food Technology, Safety and Health, Food structure and Function Research Group, Faculty of Bioscience Engineering, Ghent University, Gent, Belgium

^2^Department of Veterinary Sciences, University of Pisa, Pisa, Italy

^3^Interdepartmental Research Center Nutrafood “Nutraceuticals and Food for Health”, University of Pisa, Pisa, Italy

^4^Department of Agricultural Economics, Ghent University, Gent, Belgium

^5^China Center for Food Security Studies, Nanjing Agricultural University, Nanjing, China

^6^Charles H. Dyson School of Applied Economics and Management, Cornell University, Ithaca, United States of America

^7^Department of Food and Drug, University of Parma, Parma, Italy

*Corresponding author

E-mail: simone.mancini@unipi.it (SM)

**Consent form** English (US)

(translated into Dutch and French, Chinese, Italian and Spanish, respectively for Belgium, China, Italy, and Mexico)

You are invited to take part in a research study about the perception of edible insects as food and feed. Please read this form carefully before agreeing to take part in the study. This study is being led by *<name of researcher in charge in every country>*.

**What the study is about**

The purpose of this research is to understand consumers’ preferences for insect as feed and as food. The information from this study may benefit researchers and policy advocates in the future.

**What we will ask you to do**

You will be asked to answer several questions. The study will take approximately 10 minutes.

**Risks and discomforts**

We anticipate that your participation in this survey presents no greater risk than everyday use of the Internet.

**Compensation for participation**

You will be compensated the amount you agreed upon before you entered into the survey.

**Confidentiality and data sharing**

Your decisions during the survey will be kept confidential. All data will be recorded so that no individual participant can be identified with the results from the study. Please note that if you were recruited for this experiment via e-mail there is a chance that the information you communicated could be read by a third party. De-identified data from this study may be shared with the research community at large to advance science and health. By current scientific standards and known methods, no one will be able to identify you from the information we share.

**Taking part is voluntary**

Taking part in this study is completely voluntary. You are free at all times to withdraw.

If you have questions The main researcher conducting this study is *<name of researcher in charge in every country>*. If you have questions, you may contact *<email address of researcher>*.

**Your thoughtful answers to each question in the survey are important for us. By taking this survey, you commit to providing your honest answers to the best of your ability.**

If you consent to participate in the proposed study, please click on the “I approve” box.

- I approve and I will provide my best answers
- I do not approve

**Screening questions** English (US)

(translated into Dutch and French, Chinese, Italian and Spanish, respectively for Belgium, China, Italy, and Mexico)

**-How old are you?**

*<list from 1-99 years old>*

**-Do you consider yourself?**

- Omnivore (general food regimen eating both plants and animals)
- Vegetarian (exclusion of any meat and sometimes may also exclude by-products of animal origin)
- Vegan (exclusion from animal products, and an associated philosophy that rejects the commodity status of animals)
- Flexitarian (plant-based diet with the occasional inclusion of animal products)
- Other *<open answer>*

-**You selected vegetarian, would you consider of including insects in your diet?**

- Definitely not
- Probably not
- Might or might not
- Probably yes
- Definitely yes

(*if “Definitely not” or “Probably not” was selected*) **Could you please explain the reasons you are not considering of including insects in your diet?**

*<open answer>*

**Information treatment**

(randomly excluded)

| **Belgium** (Dutch) | Lees zorgvuldig de volgende informatie voor u verdergaat:  • De lage ecologische voetafdruk van de productie van insecten, in vergelijking met die van andere diersoorten, maakt insecten aantrekkelijk vanuit het oogpunt van ecologische duurzaamheid.  • Over het algemeen zijn eetbare insecten een goede bron van eiwitten, vetzuren, vitamines en mineralen. Dit maakt ze een potentiële voedselbron voor een gezond dieet voor mensen.  • In Europa moeten hele (onbewerkt) insecten en voedselproducten op basis van insecten voldoen aan de europeese voedselveiligheidsvoorschriften en moeten ze, voordat ze op de markt komen, door de autoriteiten worden goedgekeurd.  *Bron: Overgenomen uit FAO. 2021. Looking at edible insects from a food safety perspective. Challenges and opportunities for the sector. Rome* |
| --- | --- |
| **Belgium** (French) | Veuillez lire attentivement le texte suivant avant de poursuivre:  • Les faibles empreintes écologiques associées à la production d'insectes, compares à celles d'autres espèces animales, les rendent intéressantes du point de vue de la durabilité environnementale.  • En général, les insectes comestibles sont une bonne source de protéines, d'acides gras, de vitamines et de minéraux. Cela en fait une source alimentaire potentielle pour une alimentation humaine saine.  • En l'Europe, les insectes entiers et les produits alimentaires à base d'insectes doivent être conformes aux réglementations nationales en matière de sécurité alimentaire et, avant d'être mis sur le marché, doivent être approuvés par les autorités.  *Source: adapté par la FAO. 2021. Examen des insectes comestibles du point de vue de la sécurité alimentaire. Défis et opportunités pour le secteur. Rome* |
| **China** | 回答下面问题之前，请仔细阅读以下内容：  • 与其他家畜品种相比，昆虫产生的生态足迹较低，因此从环境可持续发展的角度来看，昆虫更具吸引力。  • 一般来说，可食用昆虫是蛋白质、脂肪酸、维生素和矿物质的良好来源。这使它们成 为健康人类饮食的潜在食物来源。  • 在中国，昆虫和昆虫类食品必须符合国家食品安全法规，并且在进入市场之前，必须 获得当局的批准。  *来源:联合国粮食及农业组织* |
| **Italy** | Leggi attentamente il seguente testo prima di continuare:  • La bassa impronta ecologica associata alla produzione di insetti, rispetto a quella delle altre specie di bestiame, li rende vantaggiosi dal punto di vista della sostenibilità ambientale.  • In generale, gli insetti edibili sono una buona fonte di proteine, acidi grassi, vitamine e minerali. Questo li rende una potenziale fonte di cibo in diete salutari per l’uomo.  • In Europa, gli insetti interi e i prodotti a base di insetti sono obbligati a rispettare le normative nazionali sulla sicurezza alimentare e, prima di essere immessi sul mercato, devono essere approvati dalle autorità.  *Fonte: Adattato dalla FAO. 2021. Looking at edible insects from a food safety perspective. Challenges and opportunities for the sector. Roma* |
| **Mexico** | Lea cuidadosamente la siguiente información antes de continuar:  • El bajo impacto ecológico asociado con la producción de insectos, comparado con otro tipo de ganado, lo hace atractivo desde el punto de vista de la sustentabilidad.  • En general, los insectos comestibles son una buena fuente de proteínas, ácidos grasos, vitaminas y minerales. Esto los hace una fuente potencial de alimentos de alta calidad.  • En Estados Unidos, los insectos para consumo humano necesitan cumplir con la regulación sanitaria antes de que puedan ser vendidos en establecimientos.  *Fuente: Adaptado de FAO. 2021.Looking at edible insects from a food safety perspective. Challenges and opportunities for the sector. Rome* |
| **US** | Please read carefully the following text before continuing:  • The low ecological footprints associated with insect production, as compared to those of other livestock species, make them attractive from an environmental sustainability standpoint.  • In general, edible insects are a good source of protein, fatty acids, vitamins, and minerals. This makes them a potential food source for healthy human diets.  • In the US, whole insects and insect-based food products are required to comply with national food safety regulations and, before entering the market, must be approved by authorities.  *Source: Adapted by FAO. 2021. Looking at edible insects from a food safety perspective. Challenges and opportunities for the sector. Rome* |

**Open questions**

**-Question 1**

| **Belgium** (Dutch) | Zou u akkoord gaan om hele (onbewerkt) meelworm (zie onderstaande afbeelding) in uw dieet op te nemen? (selecteer slechts één mogelijkheid). |
| --- | --- |
| **Belgium** (French) | Accepteriez-vous d'ajouter ver de farine entier (voir la photo ci-dessous) à votre alimentation? (sélectionnez une seule réponse et répondez en conséquence). |
| **China** | 您能否接受食用整只黄粉虫（如图所示）？（只选择一项并作相应的回答） |
| **Italy** | Accetteresti di consumare una tarma intera (vedi figura sotto) nella tua dieta? (seleziona una sola opzione e rispondi di conseguenza). |
| **Mexico** | Observe la imagen debajo. ¿Aceptaría agregar el gusano de la harina (entero) a su dieta? Seleccione sólo una opción y escriba su justificación. |
| **US** | Would you accept adding whole mealworm (see the picture below) to your diet? (select only one and answer accordingly). |

**-Answers**

| **Belgium** (Dutch) | **Belgium** (French) | **China** | **Italy** | **Mexico** | **US** |
| --- | --- | --- | --- | --- | --- |
| Ja, omdat… | Oui, parce que… | 可以，因为… | Si, perché… | Si, porque… | Yes, because… |
| Ja, maar… | Oui, mais… | 可以，但是… | Si, ma… | Si, pero… | Yes, but… |
| Misschien, als… | Peut-être, si... | 或许，如果… | Forse, se… | Quizá, porque… | Maybe, if … |
| Nee, omdat… | Non, parce que... | 不可以，因为… | No, perché… | No, porque… | No, because… |

**-Question 2**

| **Belgium** (Dutch) | Zou u akkoord gaan om in uw dieet voedselproducten (bv. repen, pasta, burgers)bereid met meelwormpoeder (zie onderstaande afbeelding) op te nemen? (selecteerslechts één mogelijkheid). |
| --- | --- |
| **Belgium** (French) | Accepteriez-vous d'ajouter à votre alimentation un aliment transformé (par exemple, une barre protéinée, des pâtes, des hamburgers) fabriqué à partir de poudre de vers de farine (voir la photo ci-dessous)? (sélectionnez une seule réponse et répondez en conséquence). |
| **China** | 您能否接受食用由黄粉虫粉（如图所示）制成的加工食品（如蛋白棒、面条、汉堡）？（只选择一项并作相应的回答） |
| **Italy** | Accetteresti di consumare nella tua dieta un cibo trasformato (es. barretta proteica, pasta, hamburger) fatto con polvere di tarma (vedi figura sotto)? (seleziona una sola opzione e rispondi di conseguenza). |
| **Mexico** | Observe la imagen debajo. ¿Aceptaría agregar a su dieta productos procesados a base de polvo de gusano de la harina (ej. barras protéicas, pasta, hamburguesas)? Seleccione solo una opción y escriba su justificación. |
| **US** | Would you accept adding a processed food (e.g., protein bar, pasta, burgers) made out of mealworm powder (see the picture below ) to your diet? (select only one and answer accordingly). |

**-Answers**

| **Belgium** (Ducth) | **Belgium** (French) | **China** | **Italy** | **Mexico** | **US** |
| --- | --- | --- | --- | --- | --- |
| Ja, omdat… | Oui, parce que… | 可以，因为… | Si, perché… | Si, porque… | Yes, because… |
| Ja, maar… | Oui, mais… | 可以，但是… | Si, ma… | Si, pero… | Yes, but… |
| Misschien, als… | Peut-être, si... | 或许，如果… | Forse, se… | Quizá, porque… | Maybe, if … |
| Nee, omdat… | Non, parce que... | 不可以，因为… | No, perché… | No, porque… | No, because… |

**Demographics** English (US)

(translated into Dutch and French, Chinese, Italian and Spanish, respectively for Belgium, China, Italy, and Mexico)

**-How old are you?**

- 18-24
- 25-34
- 35-44
- 45-54
- 55-64
- 65+

**-What is your gender?**

- Male
- Female
- Prefer not to say

**-In which state/region do you currently reside?**

| **Belgium** | **China** | **Italy** | **Mexico** | **US** |
| --- | --- | --- | --- | --- |
| Wallonia: Henegouwen, Waals-Brabant, Namen, Luik, Luxembourg (1) | North: Beijing, Tianjin, Hebei, Shanxi, Inner Mongolia (1) | Northwest: Liguria, Lombardia, Piemonte, Valle d'Aosta (1) | North: Baja California, Baja California Sur, Coahuila, Chihuahua, Durango, Nayarit, Nuevo Leon, Sinaloa, Sonora, Tamaulipas, Zacatecas (1) | South: Alabama, Arkansas, Washington, Delaware, Florida, Georgia, Kentucky, Louisiana, Maryland, Mississippi, North Carolina, Oklahoma, South Carolina, Tennessee, Texas, Virginia, West Virginia (1) |
| Flanderen: Antwerpen, Limburg, Oost-Vlaanderen, Vlaams-Brabant, West-Vlaanderen (2) | Northwest: Shan'xi, Gansu, Qinghai, Ningxia, Xinjiang (2) | Northeast: Emilia-Romagna, Friuli-Venezia Giulia, Trentino-Alto Adige, Veneto (2) | Center/Center-North: Aguascalientes, Colima, Ciudad de México, Estado de México, Guanajuato, Hidalgo, Jalisco, Michoacán, Querétaro, San Luis Potosí (2) | West: Alaska, Arizona, California, Colorado, Hawaii, Idaho, Montana, Nevada, New Mexico, Oregon, Utah, Washington, Wyoming (2) |
| Brussels Region (3) | Eastern: Shanghai, Jiangsu, Zhejiang, Anhui, Fujian, Jiangxi, Shandong (3) | Center: Lazio, Marche, Toscana, Umbria (3) | South/Southeast: Campeche, Chiapas, Guerrero, Morelos, Oaxaca, Puebla, Quintana Roo, Tabasco, Tlaxcala, Veracruz, Yucatan (3) | Northeast: Connecticut, Maine, Massachusetts, New Hampshire, New Jersey, New York, Pennsylvania, Rhode Island, Vermont (3) |
|  | Northeast: Liaoning, Jilin, HeilongJiang (4) | South and islands: Abruzzo, Basilicata, Calabria, Campania, Molise, Puglia, Sardegna, Sicilia (4) |  | Midwest: Illinois, Indiana, Iowa, Kansas, Michigan, Minnesota, Missouri, Nebraska, North Dakota, Ohio, South Dakota, Wisconsin (4) |
|  | Central south: Henan, Hubei, Hunan, Guangdong, Guangxi, Hainan (5) |  |  |  |
|  | Southwest: Chongqing, Sichuan, Guizhou, Yunnan, Tibet (6) |  |  |  |
